# Antibody responses induced by *Trichobilharzia regenti* antigens in murine and human hosts exhibiting cercarial dermatitis

**DOI:** 10.1111/j.1365-3024.2008.01059.x

**Published:** 2008-11

**Authors:** L LICHTENBERGOVÁ, P KOLBEKOVÁ, P KOUŘILOVÁ, M KAŠNÝ, L MIKEŠ, H HAAS, G SCHRAMM, P HORÁK, L KOLÁŘOVÁ, A P MOUNTFORD

**Affiliations:** 1 Department of Microbiology, 3rd Faculty of Medicine, Charles UniversityPrague, Czech Republic; 2 Department of Parasitology, Faculty of Science, Charles UniversityPrague, Czech Republic; 3 Division of Cellular Allergology, Research Center BorstelBorstel, Germany; 4 National Reference Laboratory for Tissue Helminthoses, Department of Microbiology, Institute for Postgraduate Medical EducationPrague, Czech Republic; 5 Department of Biology, University of YorkYork, United Kingdom

**Keywords:** *Antibody*, *cathepsin*, *IgE*, *schistosomes*, Trichobilharzia

## Abstract

*Cercariae of bird schistosomes (genus* Trichobilharzia) *are able to penetrate the skin of mammals (noncompatible hosts), including humans, and cause a Th2-associated inflammatory cutaneous reaction termed cercarial dermatitis. The present study measured the antibody reactivity and antigen specificity of sera obtained after experimental infection of mice and natural infection of humans. Sera from mice re-infected with*T. regenti *showed a bias towards the development of antigen-specific IgM and IgG1 antibodies and elevated levels of total serum IgE, indicative of a Th2 polarized immune response. We also demonstrate that cercariae are a source of antigens triggering IL-4 release from basophils collected from healthy human volunteers. Analysis of sera from patients with a history of cercarial dermatitis revealed elevated levels of cercarial-specific IgG, particularly for samples collected from adults (> 14 years old) comparedwith children (8–14 years old), although elevated levels of antigen-specific IgE were not detected. In terms of antigen recognition, IgG and IgE antibodies in the sera of both mice and humans preferentially bound an antigen of 34 kDa. The 34 kDa molecule was present in both homogenate of cercariae, as well as cercarial excretory/secretory products, and we speculate it may represent a major immunogen initiating the Th2-immune response associated with cercarial dermatitis.*

## INTRODUCTION

Bird schistosome cercariae of the genus *Trichobilharzia* penetrate the skin of vertebrates representing either compatible (waterfowl) or noncompatible (mammals, including man) hosts. Although parasite invasion into the skin of waterfowl leads to the maturation of adult flukes, in mammals, the parasites are not able to complete their development and die at some point after infection. In humans, infection of the skin leads to an inflammatory reaction known as swimmer's itch or cercarial dermatitis, which develops after repeated contact with cercariae. The association between cercarial dermatitis in humans and exposure to avian schistosomes of the genus *Trichobilharzia* has long been recognized although the disease is now regarded as an emerging infection (1–3). The severity of dermatitis depends on various factors including the number and duration of exposures to cercariae and the host's immune status (4). The disease manifests by maculo-papulo-vesicular eruption developing between 12 and 24 h after infection and is accompanied by intensive itching and, occasionally by erythema, fever, local lymph node swelling and oedema (5,6).

The incidence of cercarial dermatitis throughout Europe is still unknown. In part, this can be explained by difficulties with laboratory confirmation of causative agent of the disease. In patients with clinical manifestation of the disease, the parasites are destroyed soon after they penetrate into the skin and, thus histological examination of biopsies does not detect the causative agent. Various techniques, such as Cercarienhüllenreaktion, complement fixation test, IFAT and ELISA (e.g., 7–10), have been used to assess the titres of specific antibodies against bird schistosome cercariae. Although they are more sensitive than skin tests, they are not species specific and they can not be performed for a differential diagnosis of cercarial dermatitis.

Bird schistosomes are thought to die soon after the penetration into the skin of noncompatible hosts, although some larvae can partially develop and under certain circumstances migrate in a manner similar to compatible hosts [reviewed in Ref (6)]. Soon after primary infection of mice, *Trichobilharzia regenti*cercariae transform to schistosomula, which escape from the skin and migrate via peripheral nerves to the host CNS. In mice, the parasites never mature and die. However, they can survive in the nervous tissue for several weeks (11,12). The transitory presence of migrating larvae leads to pathological changes in the nervous tissue (13,14), and the infection can be manifested by various neurological symptoms (11).

Schistosome larvae cause a range of immune-related events in the skin site of infection of mammalian hosts (15). Studies of *T. regenti* infection in mice revealed that primary infection leads to an acute skin inflammatory reaction characterized by the presence of neutrophils, eosinophils, macrophages and a weak infiltration by CD4^+^ lymphocytes around the invading larvae (16). Re-infection results in the development of a more intense cellular infiltration. Whereas primary *T. regenti* infection was represented by a mixed Th1/Th2 cytokine response characterized by elevation of IFN-γ, IL-12 and IL-6, multiple re-infections led to the development of Th2 polarized response with a bias towards IL-4 and IL-5 secretion. A feature of the re-infected skin was the increase in the number of tissue mast cells, some of which appeared to be degranulating. This was accompanied by a large increase in the amount of histamine and IL-4 secretion supporting the Th2/allergic nature of the immune response (16).

The antigens that stimulate the hosts’ production of antibodies (that might serve as a diagnostic tool) and/or the inflammatory response in the skin have not previously been characterized. It might be predicted that the immune reaction in the skin is caused by the presence of components of the cercarial glycocalyx and/or by molecules (peptidases and agglutinins/lectin-like proteins) released by the cercarial acetabular glands during penetration (17–19). The composition of acetabular glands is not fully known (18) but is thought to contain both cathepsin B1 and B2 (20,21). The aims of this study were therefore to describe the development of the antigen-specific antibodies after experimental infection of mice and natural infection of humans by bird schistosomes, and to identify the antigen(s) recognized by the antibody response. We also wished to determine whether antigens released by invasive cercariae caused the degranulation of human basophils that may trigger a Th2 polarized response.

## MATERIALS AND METHODS

### Parasites and experimental infections

The *T. regenti*life-cycle was maintained in laboratory reared *Radix*sp. snails (intermediate host) and ducklings of *Anas platyrhynchos*f. domestica (final hosts) as previously described (22). Freshly emerged cercariae of *T. regenti* (*n* = 1000) were used to infect C57BL/6 strain mice (females, 12 weeks old) via the exposed hind legs. Infection was performed in the dark over 1 h at room temperature (RT). Animals were re-infected with the same dose of the cercariae, on the same site, on days 10, 20, 30 after the initial infection.

### Parasite antigen preparations

Two different antigen preparations from *T. regenti*were created as follows:

*TrH antigen* (homogenate of cercariae): Cercariae were concentrated in a small volume of water, cooled to 0°C, centrifuged at 1600 *g*, and then re-suspended in a minimal volume of cold PBS. The samples were homogenized by sonication for 3 × 30 s (Vibracell-72405 100 W ultrasonicator, Bioblock Scientific) and centrifuged twice at 16 000 *g* for 10 min. The soluble supernatants were collected and either used immediately or stored at –80°C.

*Tr E/*S *antigen*(cercarial excretory/secretory products): The contents of the cercarial penetration glands were isolated by a modified method of Mikeš*et* *al*. (18). Fresh cercariae were concentrated availing of their positive phototaxis, transferred into 15 mL of PBS buffer in a Petri dish and their secretions induced by the addition of linoleic acid (0·2 µg/mL; Sigma) at RT for 1 h. Cercarial bodies were removed using paper filtration and the E/S products concentrated by ultrafiltration (Microcon YM-10; 10 000 NMWL; Amicon) at 14 000 *g* at 4°C.

### Serum samples

Mouse sera were obtained after collection of peripheral blood from the tail of C57Bl/6 mice narcotized by Rometar and Narkamon (Spofa, Prague). The samples were collected just before each infection, and subsequently on days 20, 30, 40, 60, 90 and 120 after the last infection. Human sera were obtained from a total of 58 individuals with a history of cercarial dermatitis acquired during swimming in ponds of the Czech Republic during a period of 2002–2006. The age of patients ranged from 8 to 41 years; 35 (60·34%) individuals were between 8–14 years (children) and 23 (39·66%) patients were between 15–41 years (adults). Control negative sera were obtained from patients with no history of cercarial dermatitis. All sera included in the experiments were negative for antigen-specific IgG responses to *Toxocara canis*, *Echinococcus granulosus*, *E. multilocularis*, *Fasciola hepatica*, *Trichinella spiralis*, *Dipetalonema viteae, Schistosoma mansoni* and *S. haematobium* antibodies. The sera were stored at –20°C until they were used.

### Detection of antibodies by ELISA

Immuno plates (MaxiSorp, Nunc) were coated with 0·313 µg/well TrH antigen, or 0·156 µg/well TrE/S products, both diluted in carbonate coating buffer (pH 9·6) and left overnight at 4°C. Plates were then probed for 3 h with mouse serum samples diluted 1/80 in PBS 0·05% Tween 20 (PBS-T), and then incubated for 1 h with horse-radish peroxidase (HRP)-conjugated goat antimouse IgM (1/5000), or anti-IgG1, anti-IgG2a and anti-IgG2b(1/2000, all Caltag). The reaction was visualized by addition of tetramethylbenzidine (TMB) substrate (Sigma), stopped with 1 N hydrochloric acid and read at 450 nm on Tecan Infinite M200 reader (Scholler Instruments). In some experiments, antigen-coated plates were treated with sodium periodate according to the method described by Eberl *et* *al*. (23), to determine the role of carbohydrates on antibody binding.

Total mouse IgE was measured using a sandwich ELISA: Immuno plates were coated with 2 µg/mL of purified antimouse IgE antibody (clone: R37–72, BD Pharmingen) in PBS (pH 7·2) overnight and then blocked with 1% BSA in PBS. Serum samples (diluted 1/80) and purified mouse IgE standard (clone: C48–2, BD Pharmingen; serial dilutions starting at 0·5 µg/mL) were incubated at RT for 3 h. Finally, the plates were probed with HRP-labelled goat antimouse IgE (Bethyl) diluted 1/10 000 at RT for 1 h. Binding reaction was visualized as above.

Antigen specific human IgG to TrH and TrE/S antigens and total human IgE was measured by ELISA. Briefly, immuno plates were coated with 0·18 µg/well TrH antigen and 0·22 µg/well TrE/S antigen as above. Plates were probed with human sera diluted 1/200 in PBS-T and then with swine peroxidase-conjugated antihuman IgG (Sevapharma) at 1/30 000. Binding reactions were visualized using o-phenylenediamine dichloride substrate (Sigma), stopped by addition of 0·2 N sulphuric acid and read at 450 nm using a plate reader (Dynatech). The lower level detection cutoff was set as twice the absorbance value of naïve sera. Total IgE was determined by sandwich ELISA (ALLERgen Total IgE, Adaltis) according to the manufacturer's protocol. The KIU/L (international unit per litre) was determined; children were considered positive when the total IgE level was higher than 100 KIU/L, adults when the total IgE was higher than 150 KIU/L.

### Western blotting

For immunoblotting, TrH and TrE/S (300 µg/gel) were resolved by SDS-PAGE (10% gel, nonreducing conditions; MiniProtean-3 apparatus; Bio-Rad), and electrotransferred onto Immuno-Blot PVDF membranes (Bio-Rad) at 1·5 mA/cm^2^ in 25 mm Tris, 192 mm glycine, 0·1% sodium dodecyl sulphate and 20% methanol using Semi-Dry Transfer Cell apparatus (Bio-Rad Laboratories). Following blocking of nonspecific binding sites on the PVDF membrane with 5% nonfat milk (Bio-Rad) in PBS-T, vertical strips were incubated with mouse sera and with secondary antibodies (HRP-labelled goat antimouse IgG1 or goat antimouse IgE, both diluted 1/2000 in PBS-T); sera were diluted 1/60 or 1/25 in PBS-T for detection of Ag-specific IgG1 or IgE, respectively. Opti-4CN Substrate Kit (Bio-Rad) was used for colourimetric detection of the reactions. Alternatively, for the detection of human serum antibodies, nonspecific binding sites were first eliminated with blocking buffer (5% nonfat milk (Bio-Rad), 3% BSA (Bioveta) and 50 µm E64 (*trans*-Epoxysuccinyl-L-leucylamido(4-guanidino) butane; Sigma-Aldrich) in TRIS-T for at least 3 h. The strips were then incubated with human serum samples diluted 1/20 in blocking buffer and incubated with swine peroxidase-conjugated antihuman IgG diluted 1/500 in blocking buffer. The reaction was visualized with 3,3^′^-diaminobenzidine tetrahydrochloride (Sigma) in TRIS-T buffer with H_2_O_2_.

### 2D-electrophoresis

Samples of TrH antigen (200 µg/strip of membrane) were reduced and denatured in Rehydration/Sample Buffer (Bio-Rad; 8 m urea, 2% CHAPS, 50 mm DTT, 0,2% Bio-Lite 3/10 ampholyte, 0.001% Bromphenol Blue) were applied to linear pH 3–10 ReadyStrip IPG strips (7 cm; Bio-Rad). Rehydrated IPG strips were transferred to a Bio-Rad Protean IEF focusing cell and isoelectric focusing (IEF) performed. Prior to running the second dimension, the IPG strips were equilibrated in SDS-PAGE Equilibration Buffer (6 m urea, 50 mm Tris-HCl – pH 8·8, 20% glycerol, 2% SDS, 65 mm DTT, grain of Bromphenol Blue) two times for 20 min. After equilibration the strips were laid onto the SDS-PAGE gels and the proteins separated according to their molecular weight and then transferred onto Immuno-Blot PVDF membranes (Bio-Rad) as was described above.

### Purification and stimulation of human basophils

Basophils were isolated from the peripheral blood of healthy (nonallergic) human volunteers according to the three-step procedure described by Haisch *et* *al*. (24). This procedure included a Ficoll density gradient step, followed by counterflow elutriation and negative selection using magnetic cell sorting (MACS basophil isolation kit from MiltenyiBiotec, Bergisch Gladbach, Germany). For assessment of viability and purity of the cells, Trypan Blue exclusion and May-Gruenwald staining of cytospins were used, respectively. Purified basophils were then diluted in Iscove's Modified Dulbecco's Medium supplemented with 100 U/mL penicillin G, 50 µg/mL transferrin, 5 µg/mL insulin, 100 µg/mL streptomycin, 10% FCS to a final density of 0·025 × 10^6^ cells per ml at 37°C. Stimulation was performed in round-bottomed 96-well microtitre plates (Nunc) at 37°C and 6% CO_2_. IL-3 (2·5 ng/mL; a kind gift from Kirin Company, Gunma, Japan) was added to all wells to enhance IgE-mediated IL-4 production by basophils. Basophil cultures were incubated with TrH and TrE/S overnight; ionomycin (2 µM) and polyclonal goat anti-IgE (50 ng/mL; Biosource) served as positive controls. After stimulation, culture supernatants were collected for the measurement of IL-4 by sandwich ELISA. Briefly, IL-4 release was measured on flat-bottomed 96-well microtitre plates (Maxisorp F96, Nunc) by the Eli-Pair assay (Diaclone) according to the manufacturer's protocol.

### Statistical analysis

Statistical analyses of mouse sera were performed using Student's *t*-test and values of *P* < 0·001, *P* < 0·01, and *P* < 0·05 were considered significant. Data are the mean of a minimum of four to five samples per time point. Correlations between levels of antigen-specific IgG and total IgE in human sera were derived using the Spearman rank correlation test.

## RESULTS

### *T. regenti* infection induces IgM, IgG1 and IgG2b antibodies in mice against cercarial antigens

**Figure 1:**
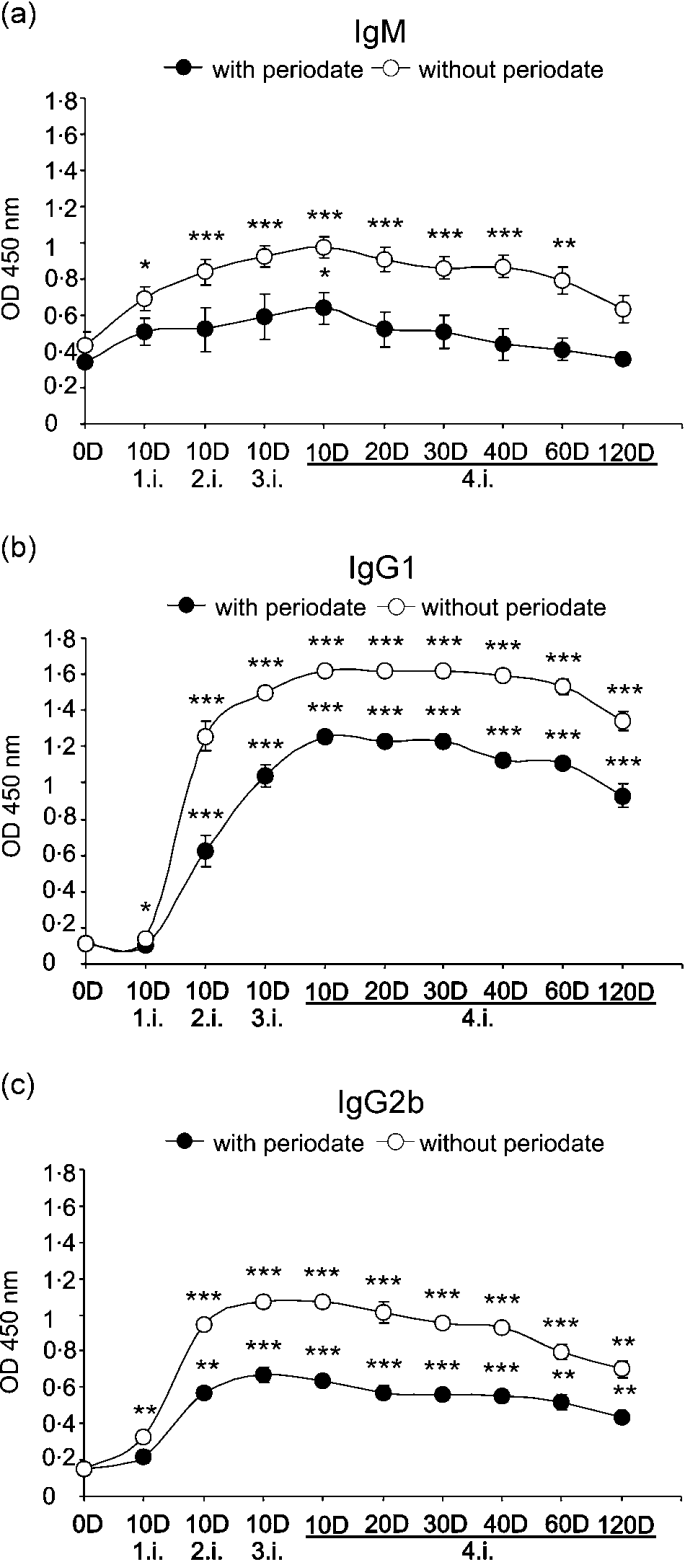
*Exposure of mice to* T. regenti *causes the production of cercarial-specific IgM, IgG1 and IgG2b antibodies*. IgM (a), IgG1 (b), and IgG2b (c) antibodies against *T. regenti* cercarial homogenate (TrH) in the sera of mice exposed to 1–4 infections. The binding of antibodies to carbohydrate structures was determined following treatment of TrH with sodium periodate (-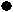-); versus control mock-treated antigen (-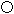-). Data points are the mean ± SEM for five mice. Statistical significances (*P < 0.05; **P < 0·01; ***P < 0·001) are relative to naïve mice (day 0).

Exposure of mice to a single dose of infective *T. regenti* cercariae induced a significant increase in the level of anti-TrH IgM antibodies detected at 10 days after infection (Figure 1a). The level increased progressively after each infection to reach a peak on day 10 after the 4th infection with the OD being 2·26-fold greater than in naïve mice. In the absence of further infection, there was a slow decline in level of IgM such that 120 days after the last infection the amount of IgM was not significantly elevated compared with that in naïve mice. Treatment of TrH antigens with sodium periodate revealed that a substantial component of the IgM response is directed against carbohydrates since at only one sampling time (day 10 after the 4th infection; *P* < 0·05) are there significantly elevated IgM antibodies against periodate-treated TrH antigens (Figure 1a).

A significant increase in the level of anti-TrH IgG1 antibodies was detected only after the second infection, and there were further increases after the 3rd and 4th infections (Figure 1b). Antigen-specific IgG1 antibodies remained elevated over the remaining time course despite there being no further exposure to cercariae and the level at day 120 was still significantly greater (*P* < 0·001) compared with naïve mice (11·5-fold greater). Similar to the profile of IgG1, levels of TrH specific IgG2b antibodies increased only after the 2nd exposure and they remained elevated after the 3rd and the 4th infections with a slow progressive decline over the remaining time course (Figure 1c). No antigen-specific IgG2a antibodies were detected (data not shown). Periodate treatment of TrH reduced the binding of both IgG1 and IgG2b antibodies but only by approximately 29·7% (IgG1) and 30·3% (IgG2b) (Figure 1b,c) showing that the majority of IgG responses are against proteins.

**Figure 2:**
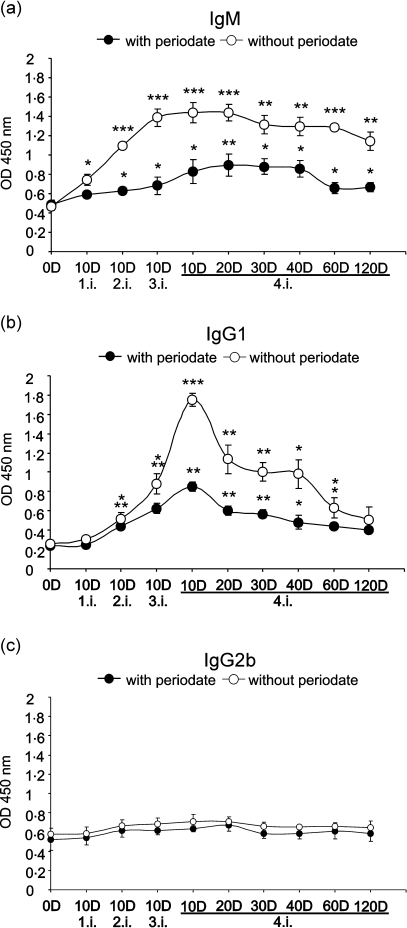
*Exposure of mice to* T. regenti *causes the production of IgM, IgG1 and IgG2b antibodies reactive against cercarial E/S products*. IgM (a), IgG1 (b), and IgG2b (c) antibodies against *T. regenti* cercarial ES products (TrE/S) in the sera of mice exposed to 1–4 infections. The binding of antibodies to carbohydrate structures was determined following treatment of TrH with sodium periodate (-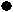-); versus control mock-treated antigen (-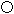-). Data points are the mean ± SEM for five mice. Statistical significances (*P < 0·05; **P < 0·01; ***P < 0·001) are relative to naïve mice (day 0).

The amounts of TrE/S-specific IgM antibodies increased significantly after each successive infection to reach a maximum 10 days after the 3rd infection (Figure 2a). However, there was little change by day 10 after the 4th infection and although there was a slight decline throughout the remaining time period, the level at day 120 was still significantly elevated compared with naïve mice (*P* < 0·01). Similar to the IgG1 response to TrH antigens, the IgG1 response to TrE/S antigens was delayed, with little increase detected after the 1st infection in contrast to the response after subsequent infections (Figure 2b). Although there was a defined peak of IgG1 antibodies to TrE/S antigens after the 4th infection, the reaction declined progressively to near naïve levels by day 120. On the other hand, there was no substantive anti-TrE/S response detected amongst the IgG2b (Figure 2c) and IgG2a isotypes (data not shown). Treatment of TrE/S antigen preparation with periodate revealed that the majority of the IgM response was targeted against carbohydrate epitopes, although, a significant antipeptide response was detected shortly after the 2nd infection and thereafter out to day 120 (Figure 2a). The IgG1 response was also directed partially against carbohydrate epitopes (Figure 2b).

### Infection of mice with *T. regenti* induces the production of total IgE

**Figure 3:**
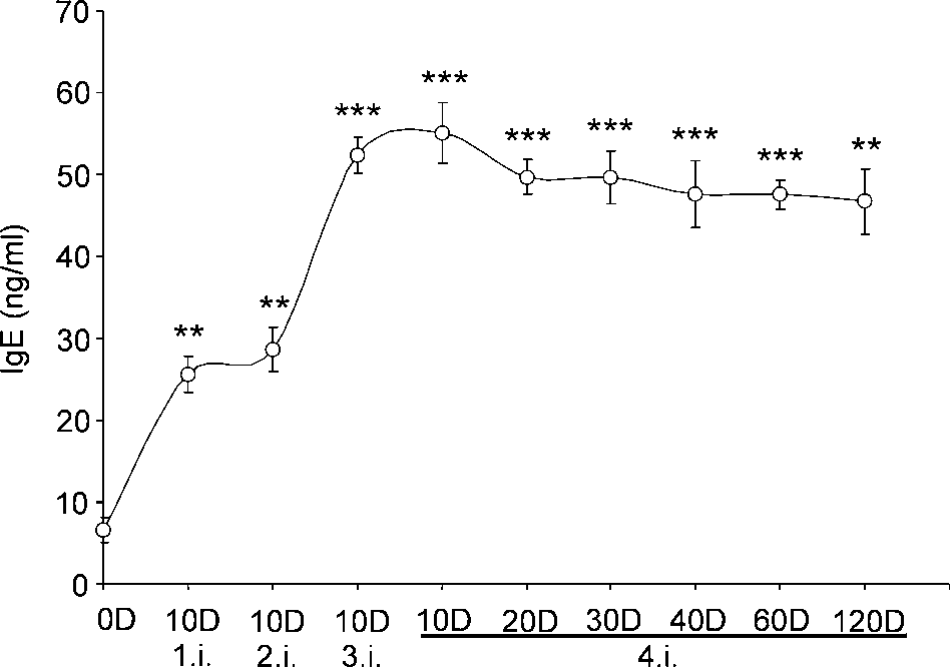
*Infection of mice with* T. regenti *leads to the elevation of total IgE*. Total IgE in serum after infection with *T. regenti* were quantified by IgE specific ELISA relative to a known IgE standard. Data points are the mean ± SEM for five mice. Statistical significances (*P < 0·05; **P < 0·01; ***P < 0·001) are relative to naïve mice.

Exposure of mice to *T. regenti* led to a significant elevation (3·8-fold increase) of the total serum IgE within 10 days (Figure 3). There were further increases, particularly after the 3rd infection, but thereafter, the levels of IgE remained fairly stable at a plateau of approximately 50 ng/mL, which is ∼7·5-fold greater than in naïve mice (all *P* < 0·001, except day 120, *P* < 0·01).

### Western blot analyses show that mouse IgG1 and IgE antibodies specifically recognize a 34-kDa antigen of *T. regenti*

**Figure 4:**
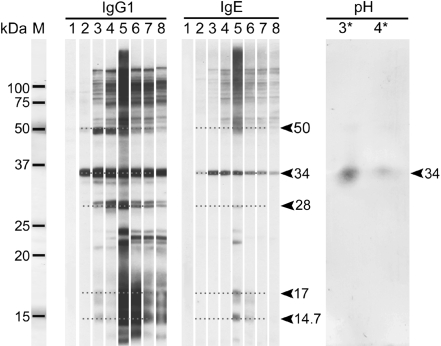
*The sera of infected mice recognise specific proteins of* T. regenti. Identification of TrH antigens by IgG1 (left panel) and IgE (centre panel) antibodies from mice exposed to *T. regenti*. Lane M represents markers; lane 1, naïve serum; lane 2–8, samples from different time after infection – lane 2, 10 days after 1st infection; lane 3, 10 days after 2nd infection; lane 4, 10 days after 3rd infection; lane 5, 10 days after 4th infection; lane 6, 20 days after 4th infection; lane 7, 60 days after 4th infection; lane 8, 90 days after 4th infection. The arrows indicate proteins of 50 kDa, 34 kDa, 28 kDa, 17 kDa and 14·7 kDa specifically recognised by IgG1 and IgE antibodies. The analysis of IgG1 reaction with TrH antigen on 2-D membrane (right panel) confirmed specific antibody binding to 34 kDa antigen. IgG1 antibodies in the serum of mouse 10 days after 4th infection recognised the 34 kDa antigen with pH optimum of approximately 3 and 4.

**Figure 5:**
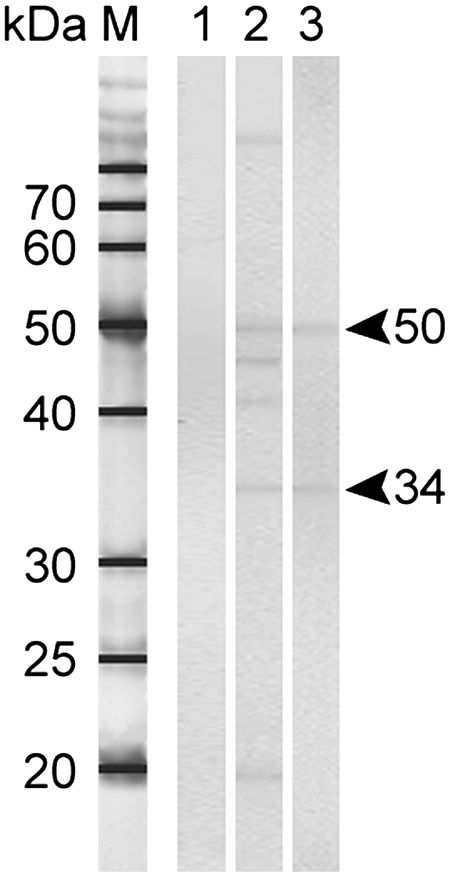
*Identification of specific protein of* T. regenti *cercarial E/S products*. IgG1 (lane 2) and IgE (lane 3) antibodies from mouse infected four times by *T. regenti* specifically recognised proteins of 50 kDa and 34 kDa. Lane M represents markers; lane 1, naïve serum; lane 2 and 3, serum from mouse 10 days after 4th infection.

Anti-sera from mice infected with *T. regenti*were tested against TrH antigens by Western blot analysis. IgG1 and IgE antibodies from re-infected mice specifically and strongly recognized a 34-kDa protein band and weakly two protein bands of lower molecular weight (17 and 14·7 kDa) (Figure 4). IgG1 antibodies also bound to proteins of about 28 and 50 kDa. The intensity of antigen recognition increased with the number of re-infections and although the level of antibody binding declined, recognition of the 34 kDa antigen was still detected 90 days after the last infection(lane 8). These results were confirmed on 2-D membrane where IgG1 specifically recognized spots of 34 kDa and of approximate p*I* 3 and 4 (Figure 4). Western blot analysis of TrE/S antigens probed with sera of four times infected mice showed that the IgG and IgE antibodies also bound to the 34 kDa protein and to the 50 kDa protein, although the intensity of binding was relatively weak (Figure 5).

### TrH and TrE/S antigens were recognized by IgG antibodies in serum samples of patients with a history of cercarial dermatitis

A total of 39 out of 58 patients with a history of cercarial dermatitis were judged to have positive titres of IgG antibodies against TrH antigens. Of these, 19 out of 23 adults (82·61%) were positive, whereas only 20 out of 35 children (57·14%) recognized TrH (Table 1). In contrast fewer patients (29/56) had a positive IgG reaction against TrE/S antigens; this comprised 15 out of 22 adults (68·18%) and only 14 out of 34 children (41·18%).

**Table 1:** Summary of results obtained by ELISA examination of human sera of total 58 patients of different age with a history of cercarial dermatitis

	Patients with a history of cercarial dermatitis				
	Age 8–14 years			Age 15–41 years	
	No. examined		No. positive (%)^a^	No. examined	No. positive (%)^a^
IgG	Tr H	35	20 (57·14%)	23	19 (82·61%)
	Tr E/S	34	14 (41·18%)	22	15 (68·18%)
IgE	Tr H	27	0	16	0
	Tr E/S	27	0	16	0
Total IgE amount KIU/L	32		21 (65·63%)	22	4 (18·18%)
			≥ 100		≥ 150

a Serum was considered as positive when its value was higher than value of cutoff (2× extinction of negative serum).

### Human IgE responses to TrH and TrE/S antigens

None of the tested serum samples either from children or from adults, had an IgE extinction value higher than the lower cutoff value in the reactions with the both antigens (Table 1). However, in respect to the extinction value of negative sera, we observed elevated antigen-specific IgE in the sera of both patient groups. In the study of TrH antigen, where the extinction value of the negative serum was 0·185, only one out of 27 children (3·70%) and 0 out of 16 adults had elevated levels of TrH-specific IgE. In contrast, the extinction value of negative serum against TrE/S antigens was 0·194 resulting in 10 out of 27 (37·04%) children's samples being positive for TrE/S-specific IgE, and two out of 16 (12·5%) adult samples being positive. These results suggest that TrE/S antigens are more important for the development of IgE-mediated allergic responses during the development of cercarial dermatitis. Quantification of total IgE in the human samples showed elevated levels in a total of 21 out of 32 children (65·63%) but only four (18·18%) out of 22 adults examined.

Our study revealed that 12 out of 15 (80%) children with elevated IgG to TrH have total IgE ≥ 100 KIU/L and 10 (73·33%) have elevated IgG to TrE/S. Nevertheless, we did not find any positive correlation between antigen-specific IgG levels and levels of total IgE. In sera of four adults positive for IgG to TrH, the levels of total IgE were also elevated (≥ 150 KIU/L), while elevated levels of IgG to TrE/S were found in two serum samples. In the case of adult patient, the levels of total IgE were related to the level of anti-TrH IgG (*r*_s_ = 0·46; *P* = 0·031), and to anti-TrE/S IgG antibodies (*r*_s_ = 0·504; *P* = 0·017).

### Human IgG antibodies specifically recognize a 34-kDa antigen of *T. regenti*

**Figure 6:**
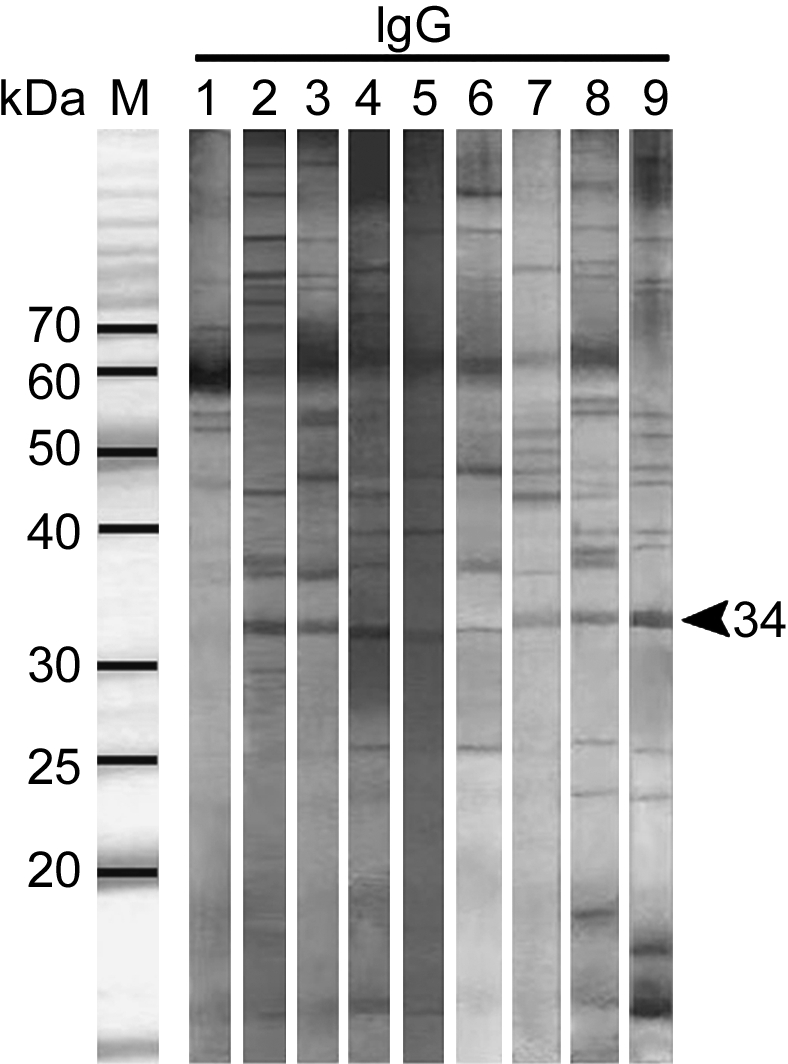
*Western blot of human antisera IgG reactivity against TrH antigens*. The specific recognition of an antigen of 34 kDa by IgG antibodies from patients with a history of cercarial dermatitis were identified. Lane M represents markers; lane 1, negative serum, lanes 2–9 sera of patients with history of cercarial dermatitis.

**Figure 7:**
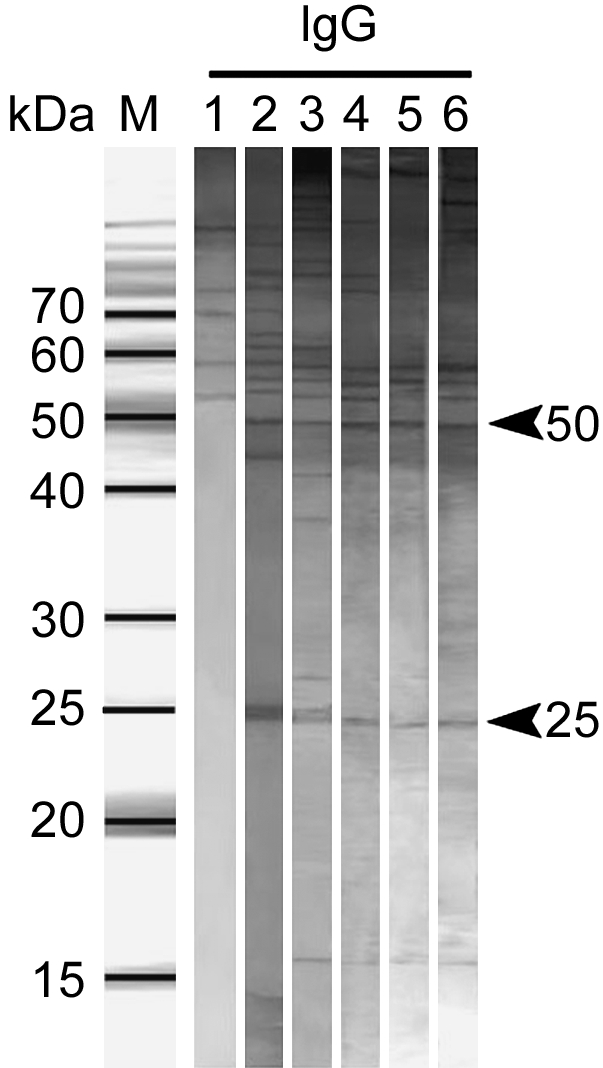
*Western blot of human antisera IgG reactivity against TrE/S antigens*. The specific recognition of 25 kDa and 50 kDa antigens by IgG antibodies from patients with a history of cercarial dermatitis were identified. Lane M represents markers; lane 1, negative serum, lanes 2–6 sera of patient sera.

In the sera of patients with a history of cercarial dermatitis, binding of IgG antibodies to TrH and TrE/S was observed only in those samples, which were judged positive by ELISA (Figures 6 and 7). However, unlike the antibody response by serum samples from an inbred strain of mice, the recognition of TrH by human IgG antibodies was quite heterogenous with many additional bands representing different proteins being recognized. Nevertheless, most serum samples specifically recognize a protein of approximately 34 kDa.

### Human basophils stimulated with TrH and TrE/S release IL-4

**Figure 8:**
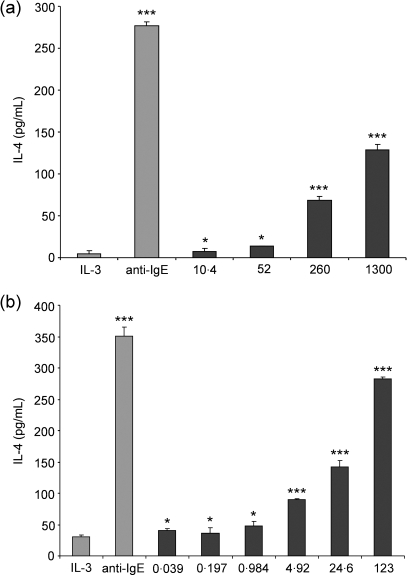
T. regenti *cercarial homogenate and E/S products dose-dependently trigger IL-4 release from human basophils*. Basophils stimulated with various concentrations of TrH (a) and TrE/S antigen (b) release IL-4 in a dose-dependent manner. All stimulations were performed in the presence of IL-3 (2·5 ng/mL). Stimulation of basophils with IL-3 and anti-IgE was performed as negative and positive control, respectively. Statistical significance related to negative control (IL-3): *P < 0·05; **P < 0·01; ***P < 0·001.

Purified basophils were stimulated with different concentrations of TrH antigens, leading to a dose-dependent release of IL-4 (Figure 8a). Doses of 10·4 µg and above induced significantly enhanced production of IL-4 compared with the negative control of IL-3, and there was weak further enhancement for doses between 260 and 1300 µg/mL. TrE/S antigens also showed ability to stimulated the release of IL-4, again in a dose-dependent manner (Figure 8b), with the highest level of stimulation resulting from the addition of 123 µg/mL TrE/S.

## DISCUSSION

Our study charts the development of antibodies against cercarial antigens of *T. regenti* in the experimentally infected murine host, and is the first to measure the humoral response in human patients with clinical expression of cercarial dermatitis. It also identifies several antigens released from cercariae, notably one of 34 kDa that might serve as a diagnostic tool.

Mice infected with *T. regenti* demonstrated an exposure-related increase in Th2–associated antigen-specific IgG1 and total IgE serum antibodies. This confirms previous observations that Th2 responses dominate after repeat *Trichobilharzia*infection and these may be largely responsible for killing schistosomula in murine host skin (16). During the early phase of *T. regenti* primary infection, the IgM antibody response was targeted largely against (periodate-sensitive) glycan structures of cercariae and their E/S products. This response is likely to be directed against components of the cercarial glycocalyx, which protects the free-swimming cercariae, but may also be directed against glycoproteins released from the pre- and post-acetabular glands. Indeed, previous studies on *T. szidati* (17) showed that both the glycocalyx and cercarial penetration glands are rich in various carbohydrates. The rapid increase in antibody responses against cercarial antigens (both TrH and TrE/S) mirrors that seen in chimpanzees exposed to multiple doses of irradiated *Schistosoma mansoni* cercariae (23). Glycans released by schistosome cercariae may also have significant effects upon the innate immune response and specifically may modulate the development of adaptive responses including antibody production (25,26)

Elevated levels of both antigen-specific IgG1 and IgG2b were observed in our study after the first infection indicating a mixed Th1/Th2 antibody response. However, with repeat infection the IgG1 response continued to increase, in contrast to Th1-associated IgG2b, supporting the progression to a Th2 dominated response (16). Repeat infection of mice also resulted in a significant increase in the total serum IgE level reaching a maximum 10 days after the 4th infection when an 8·3-fold increase compared with naïve mice was noted. The time course of IgG1 antibodies against TrE/S antigens and those from homogenized cercariae (TrH) had different profiles of development. Antibodies against TrE/S antigens increased slowly 10 days after the 2nd infection and reached a peak on day 10 after the 4th infection before declining quickly. In contrast the response against TrH antigens increased rapidly after the first infection and was maintained for a long period after the 4th infection. The E/S products of *T. regenti* are released only by living parasite during its penetration and migration through the host tissue (18). In contrast, TrH antigens will also include many abundant somatic constituents, which are likely to be present over a sustained period from flukes migrating through host tissues, or from dying worms under immune attack. Indeed, it is known that *T. regenti* larvae can be detected in mouse skin for a period of at least one week (16,27) or in nerve tissues for even longer (12) representing a continued source of antigen.

Examination of sera from patients with a history of cercarial dermatitis revealed that TrH and TrE/S antigens were recognized by IgG antibodies in the majority of sera examined, although the number of sera recognizing TrE/S antigens was lower in comparison with TrH. As above, this might be explained by the limited amount of E/S products released by larvae during penetration. The majority of sera that specifically recognized TrH and TrE/S antigens, originated from patients older than 15 years. Although we have no detailed information on the patients’ history of exposure (namely on the number of exposures to water containing infective larvae of bird schistosomes, or the number of penetrating cercariae), the higher antibody levels in the adults confirm that the antibody response is stimulated by the repeated contacts with the cercariae, which commonly occur in the field (27,28). Contrary to expectations, we were unable to detect human antigen-specific IgE responses against TrH, or TrE/S, although this may be because of competing (blocking) antibodies or other classes such as IgG4. Unfortunately, we were not able to purify the IgE antibodies due to limited availability of sera. Nevertheless, because helminth infections are associated not only with high levels of antigen-specific IgE, but also with elevation of total IgE antibodies (29), the amount of total IgE was measured in tested sera. The results showed that the elevation of total IgE positively correlated with level of antigen-specific IgG antibodies in sera of adult patients.

Despite the lack of elevated levels of antigen-specific IgE in human patients, we reasoned that the elevated levels of skin mast cells (16) and high titres of IgE in re-infected mice may implicate cells of the basophil/mast cell lineage in the development of Th2-associated responses following *Trichobilharzia*infection. Like mast cells, basophils release a number of mediators such as histamine and IL-4 after antigen-specific cross-linking of the surface FcɛRI receptors (30), which promotes allergic Th2-associated immune responses (31,32). Basophils are also important components of the human immune response to helminth infections that are associated with high levels of IgE (33,34). However, basophils can be also activated in nonsensitized humans by parasite antigens (31). In our study, both TrH and TrE/S induced basophils from healthy nonsensitized donors to degranulate and release IL-4. The consequent release of IL-4 was dose-dependent and TrE/S antigen was a more potent inducer of IL-4 release than TrH antigen. Interestingly, similar results were obtained by Machado *et* *al*. (35) who showed E/S products from *S. mansoni* cercariae to be effective stimulators of *in vitro* mast-cell degranulation and the production of histamine and IL-4. Basophils from parasite naïve human donors also released IL-4 in response to helminth products from *S. mansoni* egg antigen (36,37) and *Echinococcus multilocularis*metacestodes (38). Stimulants of basophil activation may contain peptidases, indeed, many peptidases have been identified as allergens (39) and molecular modelling shows that *Schistosoma* peptidases (e.g. cathepsin L) share epitopes with the allergen *Der P-*1 from house dust mite (40). Combined our data support the view that E/S products released upon repeated or chronic infection with schistosome helminths have the ability to activate host basophils that in turn are important in the development of Th2-polarized acquired immune responses.

Cercarial E/S products, particularly peptidases, are essential for parasite penetration through the host skin and for shedding of the glycocalyx. Studies on *S. mansoni* indicate that secretions from the post-acetabular glands may initiate a specific antibody response and serine peptidases produced by cercarial acetabular glands facilitate penetration through the host skin by degradation of the dermal and epidermal macromolecules and extracellular matrix proteins (41–43). Cysteine peptidases identified from cercarial gland contents of *T. regenti*, namely cathepsin B, also play a major role in the hydrolysation of skin proteins during penetration into the host (21). Such molecules are likely to be major immunogens capable of stimulating strong antibody responses. In this context, Western blots of TrH antigen probed with sera from re-infected mice identified several antigens (14·7, 17, 28, 34 and 50 kDa) that were specifically recognized by both IgG and IgE antibodies. The intensity of antibody binding to the antigens increased following repeat infection and the strongest IgG and IgE reaction was observed on day 10 after the last infection, corresponding with our ELISA results of antibody reactivity. The 34 and 50 kDa antigens were also both specifically identified in cercarial gland secretions (TrE/S). Previous studies of *T. regenti* cysteine peptidases identified a 33-kDa cathepsin B in cercarial extract, as well as in schistosomula (21,44) and the 34 kDa antigen recognized on our Western blots could be the same cathepsin B. We are currently investigating the identity of the 34 kDa molecule as a major immunogen using molecular and proteomic approaches, and wish to determine whether this molecule is responsible for stimulating mast cell degranulation.
